# Usability of Videoconferencing for Physical Exercise Interventions in Older Adults: Scoping Review

**DOI:** 10.2196/65552

**Published:** 2025-09-18

**Authors:** Louise Robin, Stéphane Mandigout, Charles Sebyio Batcho, Axelle Gelineau, Benoit Borel

**Affiliations:** 1 Laboratoire HAVAE - Université de Limoges, 123 avenue Albert Thomas, Limoges Cedex 1, 87060, France, 33 555457632; 2 Center for Interdisciplinary Research in Rehabilitation and Social Integration (Cirris), Québec, QC, Canada; 3 School of Rehabilitation Sciences, Faculty of Medicine, Laval University, Québec, QC, Canada

**Keywords:** usability, videoconferencing, tele-exercise, older adult, physical exercise, older adults

## Abstract

**Background:**

Videoconference combines the convenience of home-based physical exercise (PE) with group interaction and supervision of exercise in the community and could be useful for facilitating PE practice among older adults.

**Objective:**

This scoping review aims to assess the evidence regarding the usability of videoconferencing technologies. Specifically, it explores whether tele-exercise solutions based on videoconferencing are usable for older adults and professionals, and how the heterogeneity of evaluation tools influences the generalization of results.

**Methods:**

Electronic searches were conducted in databases Pubmed-Ovid Medline, Science Direct, Scopus, and CINAHL from January 2000 to March 2025 for identifying articles on measures of usability (ie, effectiveness, efficiency, and satisfaction of technology).

**Results:**

A total of 14 studies out of 2506 unique records were included. The results showed that videoconferencing strategies can effectively deliver synchronous exercise interventions. However, their effectiveness, efficiency, and user satisfaction vary depending on the technological medium used. Despite widespread use among older adults, there is a notable gap in studies evaluating usability, particularly regarding remote PE interventions.

**Conclusions:**

The review highlights that videoconferencing can be an effective tool for delivering synchronous exercise interventions to older adults. However, the effectiveness and user satisfaction depend on the technology used and the individual’s characteristics. Further studies using standardized evaluation tools are needed to better assess usability. These findings underscore the importance of continued research to evaluate the effectiveness and acceptability of videoconferencing technologies for exercise interventions and to develop customized solutions to optimize the efficacy of these interventions for this population.

## Introduction

The practice of physical exercise (PE) is defined as planned, structured, and repeated physical activity performed to improve or maintain regular physical fitness [1]. It limits some of the detrimental effects of aging and helps preserve the quality of life and independence of older adults [2], defined as individuals aged 65 years and older [3].

In particular, multimodal exercises (eg, pilates [4], the Otago exercise program [5], or the Vivifrail program [6]) have become popular for targeting balance impairments in older adults [7]. However, older adults’ commitment to physical exercise is low [8]. It may be influenced by individual factors, such as physical limitations [9,10], motivation, and personal beliefs [8,11], or environmental factors [12], such as the difficulty of accessing the infrastructure where these programs are delivered [13].

Recently, the COVID-19 pandemic has reduced opportunities for activities among older adults and similarly limited their access to physical activity programs and exercise [14]. In this context, tele-exercise refers to the practice of physical exercises conducted remotely, guided by health care professionals or trainers, aiming to improve participants’ overall physical fitness and well-being [15]. Information and communication technologies, particularly videoconferencing, allow clinicians to offer synchronous physical activities tailored to individuals’ needs. These technologies offer the potential for new strategies such as feedback, social support, coaching, and appear to be particularly useful for increasing PE engagement [16]. To be used effectively, it appears essential that the technology matches the user’s needs (clients and physical activity professional) [17].

This consideration refers to the concept of usability. The International Organization for Standardization (ISO) described usability as “the degree to which a product can be used, by identified users, to achieve defined goals with effectiveness, efficiency, and satisfaction, within a specified context of use” [18]. According to this definition, effectiveness refers to a tool’s ability to achieve its intended objective, while efficiency corresponds to the ratio of resources to results. Satisfaction corresponds to the comfort felt by the user when using a tool [18]. Usability is therefore not a fixed property of a technology but rather depends on the characteristics, needs, and experiences of its users. As previously reported by Broekhuis et al [19], usability studies are crucial for understanding the reasons behind older adults’ acceptance or rejection of technologies, considering factors such as complexity, lack of experience, and fear of reducing human interaction.

Despite the increasing use of tele-exercise strategies over the past decade, no review has specifically addressed the usability of videoconferencing technologies used in PE interventions for older adults. While there is significant research on general barriers to telehealth adherence, few studies focus on the usability of remote PE interventions for older adults, especially in terms of their effectiveness, efficiency, and user satisfaction. Therefore, it seems essential to appraise existing evidence on the usability of videoconferencing solutions for delivering PE interventions to older adults, as the effectiveness of these interventions partly depends on the usability of the technologies used. This research area is still developing, with various types of studies and methodologies being used [20], including usability tests such as the System Usability Scale, user feedback, post-use interviews, and questionnaires, resulting in a heterogeneous body of knowledge. Based on a scoping review approach [21], we aimed to assess the evidence regarding the usability of videoconferencing technologies. Specifically, it explores whether tele-exercise solutions based on videoconferencing are usable for older adults and professionals and how the heterogeneity of evaluation tools influences the generalization of results. The following two main hypotheses will be explored: (1) Tele-exercise devices are usable in terms of effectiveness, efficiency, and user satisfaction. (2) The heterogeneity of assessment tools limits the comparability of studies, making it difficult to establish standardized recommendations for integrating these technologies into clinical practice.

## Methods

### Search Strategy

This scoping review was structured according to the PRISMA-ScR (Preferred Reporting Items for Systematic reviews and Meta-Analyses extension for Scoping Reviews) guidelines [22] (see Checklist 1). The review was conducted by 2 independent reviewers (LR and BB) using the following 4 leading databases: Pubmed-Ovid Medline, Science Direct, Scopus, and CINAHL, which are among the main databases in health and physical activity field. These 4 databases cover the different dimensions of our topic, namely biomedical, paramedical, and interdisciplinary. The following keywords were identified and combined to address the research questions: (1) The target population was older adults living in the community. The population studied in the articles did not have to present any specific health characteristic (osteoarthritis, postfall, motor, or cognitive disorders), (2) videoconference should have been used as support of intervention, (3) program of PE as a type of intervention, and (4) usability as the main purpose. Synonyms were used to maximize inclusion. Keywords and related subject headings were searched using Boolean operators. The selected studies were limited to those published between January 2000 and February 2025. No language restrictions were applied to the selected studies. The MeSH (Medical Subject Headings) terms and combinations used are listed in Table 1.

**Table 1.** Research terms.

Parameter	MeSH^a^ terms used in Pubmed-Ovid Medline, Scopus, and CINAHL	MeSH terms used in ScienceDirect
Population	(Aged people) OR (Aged adult*) OR (Aging) OR (older adult*) OR (“Older people”) OR (Elder*) OR (Seniors)	(Aged people) OR (older adult)
Method of intervention	(eHealth) OR (web-based) OR (e-Health) OR (MHealth) OR (teleconferenc*) OR (Videoconferenc*) OR (Telehealth) OR (Telerehabilitation) OR (Tele-rehabilitation) OR (Teleexercise) OR (Tele-exercise)	(Videoconferencing) OR (Teleexercise)
Type of intervention	(Physical exercise*) OR (Physical activit*) OR (Fitness exercise*) OR (Physical fitness) OR (physical exercises program)	(Physical exercise) OR (Physical activity)
Outcomes	(Usability) OR (Acceptability) OR (feasibility)	(Usability) OR (Acceptability) OR (feasibility)

a MeSH: Medical Subject Headings.

### Eligibility Criteria

The selection of studies was conducted based on prespecified PICOS (Participants, Intervention, Comparison, Outcomes, and Study design) criteria [23] (see Textbox 1):

Non–peer-reviewed conference and journal publications, review articles, and publications that took place in hospitals, rehabilitation centers, or care settings were excluded.

Textbox 1. **Eligibility criteria.**

- Participants: Older adults (aged 65 years and older) living in the community.
- Intervention: Physical exercises delivered via videoconferencing technologies. The tele-exercise should have been delivered at home or in the context of the local community.
- Comparison: A control group was not necessary for this research.
- Outcomes: Information on usability as defined by the International Organization for Standardization (ISO) criteria (even if it was not the main outcome)—different criteria such as (1) efficiency (ease of use, reliability, and cost), (2) effectiveness (retention rate, rate of sessions completed, and adherence rate) of videoconferencing technologies, or (3) level of satisfaction (feeling about technology) or acceptance of the older adults—testified to the usability of the technology as a means of remote intervention.
- Study design: We included randomized controlled trials (RCTs), nonrandomized controlled trials (NRCTs), noncontrolled trials (NCTs), crossover, and pilot studies without a control group (qualitative or quantitative design).

### Data Collection and Analysis

#### Selection of Studies

Using Covidence software (Veritas Health Innovation), 2 authors (LR and BB) reviewed and prescreened all titles and abstracts of studies identified by the search strategy for possible inclusion according to the PICOS eligibility criteria. The full text of the preselected articles was checked for eligibility.

#### Data Extraction

When articles met the eligibility criteria, we extracted, from the full text, authors’ names, year of study, country, objectives, study design, population (ie, sample size, age, and place of residence), intervention modalities (eg, technological support, group, or individual sessions) and, when available, comparator (eg, face-to-face intervention or simply counseling), results, and conclusions on usability (effectiveness, efficiency, and satisfaction) of the proposed technologies. A narrative synthesis of the data was produced [24].

## Results

### Overview

**Figure 1.**
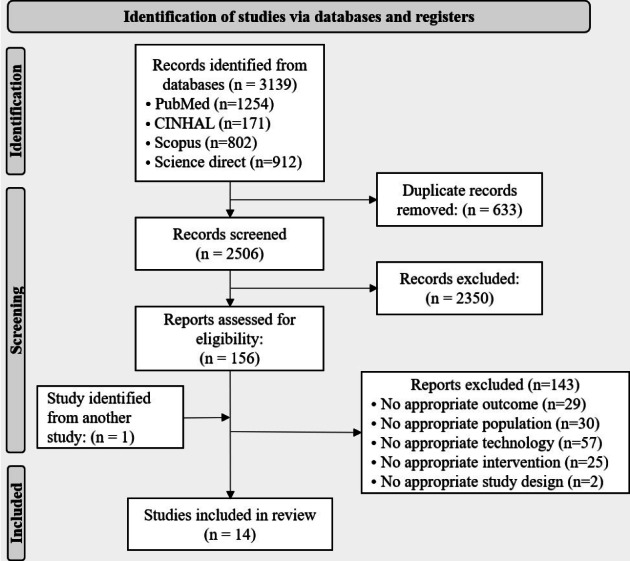
PRISMA-ScR (Preferred Reporting Items for Systematic reviews and Meta-Analyses extension for Scoping Reviews) flow diagram.

The search strategy yielded 3139 records, from which 2506 abstracts were selected after eliminating duplicates. After title and abstract selection, 156 articles were retained for full-text screening. After reading each publication, 1 article was found and added after reading an included article, and 143 publications were excluded. Figure 1 shows the reasons for article exclusion. Finally, 14 full-text publications were included (see Figure 1).

### Included Studies

The main characteristics of the studies are summarized in Table 2. The studies were published in English and came from various countries: England [25], the United States [26-32], Brazil [33], Canada [34,35], Singapore [36], Japan [37], and Israel [38].

**Table 2.** Main characteristics of the included studies.

Study	Study design and objectives	Population	Intervention, supervision, duration, and frequency of intervention technology and equipment	Outcomes
Granet et al [35], 2021	RCT^a^ to examine and compare the acceptability, feasibility, and potential benefits of 2 modalities of web-based PA^b^ interventions in older adults.	Nonphysically active community-dwelling older adults (>60 y) from the CRIUGM’s^c^ volunteer database. EG^d^: 70.7 years (SD 5.2), n=38 (81% of women); CG^e^ (recorded group): 69.6 years (SD 5.1), n=45 (83% of women).	Exercise protocol: Muscle function, flexibility, and cardiovascular exercises. A total of 55 minutes, 3 days/week for 12 weeks. Program delivered by 2 certified instructors (kinesiologists). Participant-to-professional ratio=10-14:1. Online via Zoom videoconferencing interfaces.	Initial ability to use technology in both groups: >78% used technologies for more than 1 y, >82% considered themselves “tech savvy,” and >59% used touchpad. Feasibility: Adherence rate: 85%; EG: 89%, CG: 81% Retention Rate EG: 16% drop out versus CG: 46% -> lack of interest Acceptability of intervention (4-Likert scale): Satisfaction: EG: 77% vs CG: 64% Enjoyment: 100% Perceived difficulty (easy): EG: 69% vs CG: 82% Perceived exertion (a little easy - a little difficult): EG: 42‐42% vs CG: 48‐37%. Satisfaction precautions: ie, phone close by, remove objects or carpets from path, levels of progression. No adverse events. No model or analysis grid reported.
Gell et al [30], 2022	RCT to determine the feasibility of remotely delivered exercise (tele-exercise) for older, rural cancer survivors.	Cancer survivors (>60 y), 2/3 are classified as obese. EG: 70.1 years (SD 5.3), n=20 (80% of women); CG (waiting list): 70.6 years (SD 6.2), n=19 (78% of women).	EnhanceFitness (tele-EF) exercise program, inclusive of aerobic, strength, and balance training. For 1 h, 3 days/week for 16 weeks. American Council on Exercise certified instructors. Participant-to-professional ratio: 10-12:1 online via Zoom videoconferencing interfaces.	Feasibility: Enrollment: 64% consented to participate, higher than 50% expected. Adherence rate: median 86.9% (IQR 79‐94%) Attrition: 95% completing the study. Safety: Precaution: assistant joined each class to monitor for safety No serious adverse events. No model or analysis grid reported.
Gell et al [28], 2021	Exploratory mixed methods study: (1) examine the needs of older adults previously enrolled in community-based exercise for transition to tele-exercise, (2) identify barriers to and facilitators of tele-exercise uptake and continued participation, and (3) describe technology support challenges and successes encountered by older adults starting tele-exercise.	Community-dwelling older adult with symptomatic knee osteoarthritis (>65 y). EG: 74 years (SD 6.3), n=44 (86% of women).	EnhanceFitness (tele-EF) exercise program, inclusive of aerobic, strength, and balance training. For 1 h, 3 days/week. Participant-to-professional ratio: Not reported, session available for all participants. Online via Zoom videoconferencing interfaces.	Material: 91% owned a smartphone and had broadband access, but 1/3 did not own a tablet and 1/3 did not own a computer with a webcam. Acceptability (senior Technology Acceptance Model [TAM]): Attitudinal beliefs: 8.7 (SD 3) out of 10. Control beliefs: 8.9 (SD 2.5) out of 10. Gerontechnology anxiety: 3.5 (SD 4) out of 10. Challenge during setup phase: no device camera or microphone (n=1). Challenge during classes: internet connectivity issues (n=5); Zoom audio (n=11), joining or staying on the meeting (n=15). Facilitating conditions: 48% required a single call to address the technology challenges. Adherence: median 93% (IQR 88%-98%).
Hawley-Hague et al [25], 2021	Feasibility study: Usability, acceptability, and feasibility of smartphone-based teleconferencing for health professionals and older adults.	Community-dwelling older adults. EG: 77 (range 64‐92), n=7. Health professionals: n=11.	Virtual intervention: Otagot program + balance exercises from the FaME^f^ program (evidence-based falls management exercise). For 2 wks: classic intervention before usability test. Program delivered by a health professional, 1 x/week during 2 weeks in individual and 1x in a single group session. Researchers present with participants and help to use technology if difficult. Participant-to-professional ratio: 1:1 for the individual session and 2-3:1 for the group sessions. Samsung Galaxy S4 with 4G network connected to TV or screen provided by HDMI cable or Chrome Cast. Broadband (if not available) has been set up and paid for by the research team (as a smartphone) Wireless headset. Software: Skype.	Issue log or field notes: Acceptability and Usability (TAM): Setting up and connecting the technology and accessing Skype requirement for internet or 4G. Poor connectivity -->limited reliability of teleconferencing. Positioning of the technology for delivery exercise. Anxiety and safety concerns: the patients were not as concerned as the health professionals. Cost: Broadband installation met with resistance, even when it was proposed to provide it, due to the cost that would be incurred once the study was completed. Experiences of using the technology: issues with the positioning of technology and with view and contrast. Feasibility: Ideal size of group, types of exercise that could be delivered through the smartphone.
Ho and Merchant [36], 2022	Cross-sectional study to study the acceptability of technology and perceived self-reported benefit of tele-exercise in our community-dwelling older adults.	Older adult (>60y) in community-dwelling. EG: 69.1 years (SD 4.7), n=42 (93% of women)?	Healthy Aging Promotion Program for You (HAPPY): multicomponent exercise focusing on cognition and physical function. Minimal length of 8 sessions. 3 sessions of theoretical training and cocreation of dual-task exercise and 8 hours of on-site training as assistant instructor and assessment. Program was conducted by health coaches and peer leaders who were trained to perform dual-task exercises by physiotherapists. Participant-to-professional ratio: No reported, session available for all participants. Online via Zoom videoconferencing interfaces.	Online questionnaire regarding technology Acceptability of digital technology: Utility: 91% agreed that technology is a good idea and 79% agreed that technology would allow them to be independent for longer. Ease of use: 79% thought they had the necessary knowledge and 86% thought they could use the technology if someone showed them or provided an instruction manual. A total of 41% disagreed or were neutral that technology is easy to use and that technology is easy to learn. Facilitating condition (knowledge, external assistance): Someone was available for technological assistance for 91% of participants. Technology apprehension: 7% (n=24) were apprehensive about using technology. No model or analysis grid reported.
Hyodo et al [37], 2023	Pilot study to examine the feasibility, safety, and enjoyment of a web-based aerobic dance exercise program and the usability of a web-based exercise delivery system using a videoconferencing platform for older adults.	Older adult (>65y) in community-dwelling. EG: 77.6 years (SD 4.5), n=16 (75% of women).	Slow aerobic dance exercise (SADE). Duration of 20 min, every weekday for 8 weeks. Participant-to-professional ratio: 16:1. Online via Zoom videoconferencing tablet interfaces.	Feasibility Retention rate=93.8% Adherence rate=97.40% Security: No adverse event. Enjoyment, 11-point Likert scale: improvement throughout sessions. Usability (interview) Ease of use: 27% had no problem. 73% had some challenges at the beginning. No model or analysis grid reported.
Jennings et al [26], 2020	Pilot study to describe the preliminary results and lessons learned from the rapid implementation of a telehealth-supported group exercise intervention.	Veterans (>65 y), with authorization of Durham Veterans Affairs (VA) Medical Center (offered at 17 medical centers in the United States). EG: 74.0 years (SD 6.7), n=308.	“Gerofit program”: (cardiovascular exercises, strengthening on individual machines + classes, such as Tai Chi, yoga, chair, or floor. Total of 3x/wks. For at least 8 weeks for some centers. Program delivered by 2 instructors. Participant-to-professional ratio: variable group sizes. Online via Zoom videoconferencing tablet, smartphone, or computer interfaces, or Veteran Affairs (VA) supported platforms (The VA supports telehealth using VA Video Connect (VVC) within the VA Virtual Care Manager (VCM) platform).	Feasibility Adherence rate: 6.4% attended fewer than 25% of the total available number of GTH^g^ classes, and the majority (93.6%) attended classes regularly, with 53% attending more than 75% of the time. Satisfaction and safety: 94% are likely to recommend GTH to another veteran, and 95% said they felt safe exercising at home. No model or analysis grid reported.
Patel et al [29], 2022	Pilot study to evaluate the feasibility and acceptability of remotely delivered EF (tele-EF).	Older adults (>65 y) residents of Lewis County, with symptomatic knee osteoarthritis. EG: 71.8 years (SD 5.8), n=15 (93.3% of women).	EnhanceFitness (EF): Strength training involved progressive resistance exercises using adjustable 1- to 10-pound ankle and wrist weights. EF-certified instructor 60 min, 3 days/week for 12 weeks. Participant-to-professional ratio: 10-12:1. Online via Zoom videoconferencing interfaces.	Feasibility Retention rate: 55.6% enrolled. Adherence rate: 86.7%. Acceptability of program (satisfaction): Median 91.4% (IQR 84.5‐94.3%; n=15) Material: 5 participants were loaned a cellular enabled tablet Technology acceptance (TAM scale) Ease of use: 6.4/7 Usefulness: 6.6/7 Financial cost: 2.8/7 Intention to use: 6.3/7 No model or analysis grid reported.
Pinto et al [33], 2023	Feasibility study to compare the barriers and facilitators of telerehabilitation between groups of people with and without Parkinson disease.	Older adults with and without Parkinson disease (PD). EG: 69.00 (IQR 64.68-73.32), n=14; CG with PD: 69.00 (IQR 65.17- 72.83), n=12.	Dance for PD. Total of 60 min, 2 days/weeks for 8 weeks. Professional dancer and physiotherapist sequence of movements. The participant-to-professional ratio was not reported. Online via Zoom videoconferencing tablet, smartphone, or computer interfaces.	Feasibility Retention rate: 100% Adherence rate: 91.7% to 87.5% with PD and without PD, respectively. Usability Connection: 5.8 reported poor connection. Sound problem: <2.4% in both groups. Difficulty accessing or managing between 9.6% and 10.4%. Security: Precaution: Check of environment (enough and clear space to move). No adverse event. No model or analysis grid reported.
Robin et al [34], 2024	Feasibility and pilot study. Explore the feasibility, acceptance, and usability of a PA program offered synchronously via a mobile robotic telepresence (MRP).	Older adult: EG: 83.5 years (SD 4.5), n=21.	Exercise program inspired and based on the Otago exercise program. Total of 60 min, 2 days/weeks for 4‐6 wk. Occupational therapist. Participant-to-professional ratio: 3:1. Online via the MRP.	Feasibility: Retention rate: 17/21 Adherence rate: 58% Usability: French version of system of usability scale (F-SUS): 55.0, SD 18.7, out of 100, reflecting low usability. Clinicians’ observations and semi-directive interview (UTAUT^h^ model): Effectiveness: A total of 61 sessions were offered to older adults Efficiency: (1) A total of 3 cancelations: 2 instances of impossible simultaneous connections (participants or therapist), 1 abnormal noise (possible power surge). (2) Internet connection issues (shortened or interrupted sessions). (3) A total of 3 sessions with no visibility between participants. Satisfaction: Hedonic motivation score: 4.7 (SD 1.6) out of 7.
Schwartz et al [38], 2021	Pilot study to explore the feasibility of an 8-week intervention in a group of older adults.	Older adults (>60 y old) living in the community. EG: 71.5 (SD 4), n=31 (67% of women).	Exercise protocol. Resistance and aerobic exercises; individualized program according to perceived exertion (Borg scale). 45 min, 2 x/week for 8 weeks. Search teams=2 physiotherapists +2 personal trainers, 2 instructors, and 1 technical assistant. Participant-to-professional ratio: 15:1. Online via Zoom videoconferencing interfaces. WhatsApp group chat or reminders.	Online questionnaire: Satisfaction levels with the technological aspects (final survey): Ease of use: 7 (5-7) out of 7. Quality of video and audio: 7 (5-7) and 7 (5-6) out of 7. Future intentions: 97% would like to continue. Enjoy training: 7 (5-7) out of 7. Perceived exertion: 6 (3–7) out of 10. No model or analysis grid reported.
Tomita et al [31], 2016	RCT to explore attrition rate and effectiveness to the intervention on falls, balance, and depression.	Older adults (60-90) living in the community. N=51; EG: 72.3 years (SD 7.7), n=25 (92% of women). CG: 74 years (SD 7.8), n=26 (87% of women).	EG=Virtual-Group Exercise at Home (V-GEAH). The exercise program inspired by Tai-Chi and the Otago program. CG=encouragedto walk. 25 to 40 min, 3 x/week for 24 weeks. Certified fitness trainer. Participant-to-professional ratio: 25:1. Intervention is possible on a desktop or laptop computer. Video communication software called ooVoo (ooVoo LLC) and a free program called ManyCam (ManyCam, IPM).	Feasibility: Adherence rate: 84.4%. Satisfaction: 5% of participants thought the 6-month duration was very long while 77% said they would miss the V-GEAH session. Security: Participants felt safe. No model or analysis grid reported.
VanRavenstein et al [32], 2020	Controlled study to examine the feasibility of tele-intervention to a small group of low-income older adults living independently in the community.	Older adults (>55 y), relatively autonomous (able to stand for 15 minutes, able to move 150 feet, follow instructions, charge, wear and use a Fitbit tracker). EG: 74 (60-84), n=6 (100% of women); CG: 69 (57-74), n=6 (83% of women).	Modified Otago exercise program: strength and balance exercises. EG=distance session; TG=face-to-face session; 2 x/week, 12 weeks Program physical therapist (PT), and PT student. Participant-to-professional ratio: 6:1. Instructor: desktop computer with a 32-inch screen, speakers, and the Vidyo telehealth platform. Participants could view the instructor using a second computer. Fitbit (recorded walking activity).	Qualitative interviews (Constructivist grounded theory): Feeling on the participation in an exercise program over the technology. Ease of use: difficult to the touch screen (n=2). Satisfaction: Participants appreciated the telehealth sessions.
Wu et al [27], 2006	Pilot study to assess the feasibility of a group intervention designed for balance-impaired older adults to improve their balance and reduce their fear of falling.	Older adults (>65 y) community-dwelling. EG: 81 (IQR 72-93), n=17 (76% of women).	Yang style Tai Chi Quan movements emphasizing flexibility, strength, and balance coordination. 1 h 3 x/week for 15 weeks with a Certified tai-chi teacher. Participant-to-professional ratio: 6-11:1. Video camera and ordinary TVs, displaying all participants for the instructor, and displaying either the instructor alone or with other participants.	Feasibility Adherence rate: 78 ± 15% (ranging from 51 to 98%) Drop out: n=3 Interview questionnaire and logbook Level of acceptance and satisfaction: All subjects reported favorably about the program via videoconference. Ease of use: Most subjects (n=15) reported no concerns. A total of 2 participants required assistance until the third week. Efficiency: Participants were satisfied with image and voice quality. Technical support mainly concerned connection problems, audio problems (microphone volume), or hardware problems (camera, microphone, or audio circuit failure). Most problems were solved by telephone. Hardware problems required 1-hour home visits. No model or analysis grid reported.

a RCT: randomized controlled trial.

b PA: physical activity.

c CRIUGM: Centre de Recherche de l'Institut Universitaire de Gériatrie de Montréal.

d EG: experiment group.

e CG: control group.

f FaME: Fitness and Mobility Exercise.

g GTH: Gerofit-to-Home.

h UTAUT: Unified Theory of Acceptance and Use of Technology.

#### Publication Year and Study Participants

Most of the identified articles were relatively recent as none of the included articles were published before 2006; 1 study was published in 2006 [27] and 12 between 2016 and 2023 [25-33,35-38]. Sample sizes varied across the studies, ranging from 7 to 302 participants. Participants were, on average, aged 73.1 years, with an average age range from 69.6 to 81 years, according to the sources reviewed.

#### Intervention

The interventions consisted of the adaptation of a PE program inspired by preexisting home-based activity programs, eg, Gerofit [26], Otago [25,32,34], or yoga and tai chi techniques [27] or designed by physiotherapists [38]. Except for 1 study [25], which lasted only 3 weeks, PE interventions duration ranged between 8 and 24 weeks. The interventions were supervised by health professionals (ie, kinesiologists, physical therapists, physiotherapists, and occupational therapists) or certified trainers. Furthermore, 2 studies compared the group receiving a live remote intervention with a group receiving a recorded intervention [35] or a control group receiving a traditional intervention [32]. The participant-to-professional ratio varied across studies, ranging from 1 to 25 participants, and could also fluctuate within the same study depending on the number of available participants.

#### Videoconferencing Systems

Authors used different terms to describe the intervention: teleconferencing [25], videoconferencing [27,38], tele-exercise [28,30,34,36], telehealth [26,32], web-based intervention [35,37], and virtual-group exercise [31]. Similarly, various technological supports were used: computer [31,32], tablet [37], smartphone [25], TV screen [27], and telepresence robot [34], allowing access to various videoconferencing software such as Zoom (Zoom Communications) [26,28,30,33,35-38], Skype (Microsoft) [25], WhatsApp (Meta Platforms) [38], or other undetermined applications [27,31,32,34]. Despite the different terms and configurations used, all these studies proposed a synchronous intervention with direct interaction.

### Usability of Technological Support

#### Overview

In this synthesis, various methods were identified in the included articles for assessing the usability of videoconferencing technology for delivering PE programs to older adults. This included qualitative interviews [27,32,38], self-administered questionnaires [29,34-36], and observations [25,26,30,31,33-35,37]. In addition, some studies applied theoretical frameworks such as the constructivist grounded theory [32], the Technology Acceptance Model [25,28,29], or the Unified Theory of Acceptance and Use of Technology (UTAUT) model, and system usability scale [34]. These tools and frameworks allowed for the evaluation of different aspects of the usability of videoconferencing technology, including its effectiveness, efficiency, and user satisfaction with the technological support in delivering tele-exercise programs.

#### Effectiveness of Technological Support for Videoconferencing

All studies indicate that it is possible to deliver PE interventions synchronously via videoconferencing technology. Mean adherence rate of intervention was 87.8% (range 78%-97%) [27-35,37,38]. Reasons given for continuing or not continuing exercise during studies were independent of the technology used. There were individual characteristics, like lack of interest in the intervention or health problems [35]. The instructions and progression of the exercise were the same as face-to-face [31]. All the technological supports have been effective in delivering video conferencing intervention programs.

#### Efficiency of Technological Support for Videoconferencing

A total of 12 studies evaluated the efficiency of the interventions offered by videoconferencing [25-30,33-38]. These studies report the correct efficiency of the technologies used, but certain points of concern concerning technological anxiety, ease of use and learning, technological reliability, safety, and cost are highlighted.

##### Ease of Use and Learn

Hyodo et al [37] reported that 73% of participants had some problems using technology at the beginning. VanRavenstein et al [32] highlighted that 2 participants had difficulty using the touch screen. In the study led by Granet et al [35], 69% of participants found the technology easy to use. Ho and Merchant [36] highlighted that, while 79% of respondents reported knowing to use the system, 57% (n=24) were apprehensive about using the technology. In addition, 41% (n=17) disagreed or were neutral that technology is easy to use. However, a majority (n=25, 60%) of respondents agreed with the affirmation “technology is easy to learn.” Furthermore, 86% (n=36) agreed that they could accomplish the task if someone showed them or through an instruction manual [36]. Similarly, Robin et al [34] reported that participants assessed the expected effort to use a telepresence robot at 4/7. If the technology was easy to use, the presence of technical support was appreciated.

Finally, Patel et al [29] and Schwartz et al [38] reported that participants rated ease of use at 6/7 and 7/7, respectively. For some participants, the proper use of the tool required a learning period from the intervention sessions in 2 studies [25,27]. These sessions were either 2 face-to-face sessions to learn about the technology and software [25] or a short 2.5 hours of training session [27]. In addition, to deal with problems arising during the course (staying on the meeting), telephone assistance was also necessary [28].

##### Reliability of Technology

In case of technology-related issues, the authors report that support services can assist with various aspects, including connectivity and audio adjustments (such as modifying microphone volume). They also help with troubleshooting hardware issues (eg, malfunctioning camera, microphone, or audio circuit in the videoconferencing device) that could compromise the interaction between the clinician and the participant, as well as the execution of the intervention [25,27-30,33,34,38]. The technology support chosen to be more or less effective depends on the group. Jennings et al [26] indicated that the group size was influenced by the platform used. The Veteran Affairs platform had limitations on the number of people observed simultaneously, while the Zoom platform did not.

In terms of cost, the economic situation of older adults was considered in 2 studies. For 35% of the participants in the study of Ho and Merchant [36], this aspect limited technology use. Similarly, participants (n=7) were resistant to broadband installation because of the cost that would have to be borne once the study was completed [25].

### Satisfaction of Technological Support for Videoconferencing

Some people were afraid to have the technology installed in their homes, finding it too intrusive. Gell et al [30] reported that participants were not anxious about using the technology (mean score 3.5, SD 4; out of 10). According to Hawley-Hague et al [25], the initial concerns of the older adults were about installing the equipment and using it (sometimes large screens in small spaces). Nevertheless, most of the participants managed to use the technology without major difficulties. Where difficulties arose, the use of the technology was facilitated by the existence of associated services [25,27].

However, as reported in many other studies, people were satisfied with the overall intervention, the proposed program and the technology [25-27,29,31,32,34-38]. They were willing to continue the interventions by videoconference if possible [29,31,38].

Hawley-Hague et al [25] reported that for health professionals, a display issue emerged: difficulty in seeing contrasts when the older person was dressed in black or if the room light was low. This was exacerbated in group sessions as the individual image was smaller. While these difficulties were not a concern for the patients who felt safe performing the exercises, they raised increased safety concerns according to the professionals [25]. However, no adverse events were reported by different authors [30,33,35]. Jennings et al [26] also reported that 95% of participants had reported feeling safe during exercise sessions.

Various advantages of videoconferencing technologies were noted, enhancing user satisfaction. These technologies made it possible to limit external risks such as frozen roads [27] and were more interesting than telephone follow-up, particularly during the follow-up phase [25]. The group sessions enabled by technology provided considerable added value through possible interaction between participants and the instructor. Schwartz et al [38] reported that for one person, the group activity allowed by videoconferencing helped alleviate feelings of loneliness and created a positive atmosphere. The instructor’s remote presence via videoconferencing and professionalism were appreciated and reassuring [31,34,38]. A total of 3 studies reported that the technology delivered an intervention in which participants perceived benefits on physical and mental well-being [34,36,38].

## Discussion

### Principal Findings

The use of videoconferencing allows professionals to offer PE interventions to older adults. Nevertheless, it seemed important to evaluate the usability of the technological support used to propose these interventions while encouraging the adherence of the older adults. To our knowledge, this is the first scoping review to evaluate the usability of various technological supports (ie, laptop, tablet, smartphone, or telepresence robot) for delivering tele-exercise programs to older adults via videoconferencing. Our results indicate that the usability of tele-exercise programs may vary according to the technological support used for their delivery via videoconferencing.

### Usability of Videoconferencing Technologies

All the studies included in this review demonstrated the effectiveness of technological videoconferencing media in the implementation of live and remote PE interventions. Adherence rates were variable, ranging from 57% to 100%, and were sometimes higher than adherence rates for exercise programs offered without videoconferencing [39]. The high adherence of participants in the studies testifies to the effectiveness of the technologies used. It appears possible for participants to join the interventions conducted remotely. These technologies (ie, laptop, tablet, smartphone, or telepresence robot) offer great accessibility for remote communication, thus facilitating exchanges without the need for physical presence. This accessibility is likely a key factor in the high adherence rates observed, allowing participants to engage in PE interventions from the comfort of their own home, while overcoming geographical and logistical barriers.

While videoconferencing is effective in providing a live remote PE intervention, our results indicate various points of caution. First, variations in participants’ adherence to the program could be influenced by user characteristics. It may be more difficult to use technology for adults inexperienced with technology [35]. The results of this scoping review showed that older adults may need external assistance to use the technology [25,27,30,36]. Some factors hold back the use of technology by older adult users, namely the cost of the technology, followed by the complexity to use it [40,41]. This may justify the importance for obtaining environmental supports, that is, financial support and training opportunities, as it appears essential to help older users to overcome technological barriers (ie, connection issues, audio issues) [42]. Second, variations in technology effectiveness could be influenced by device characteristics and user satisfaction with the technology. According to our results, clinicians and older adults may encounter reliability issues with the technology that compromised the intervention and thus hindered users’ satisfaction and safety of older adults [27,32]. Some platforms appear to be less usable than others for large group interventions (ie, Zoom platform on smartphone) [25,26]. For videoconferencing systems, the required bandwidth, the security of the transmitted images and the reliability of technology are important factors to consider [43]. In the event of difficulties with technology, some solutions exist, for example, Granet et al [35], who suggested that recorded sessions may be appropriate for participants who are temporarily unable to attend live, online group sessions.

### Assessment of Usability

Qualitative interviews, self-administered questionnaires, and observations were the preferred approaches for evaluating the efficiency of technological support and user satisfaction with the system and the proposed interventions. These self-reported methods provide valuable insights into users’ perceptions: when users report positive experiences with technology, they are more likely to adopt or continue using it [44]. Some studies have also based their measures on theories like constructivist grounded theory [32] or the Technology Acceptance Model [25]. However, while a study did use existing usability scales such as the System Usability Scale [45] or the Quebec User Evaluation of Satisfaction with Assistive Technology [46], the lack of widespread use of these standardized tools makes it difficult to draw general conclusions. While qualitative methods are essential for providing specific details that quantitative measures sometimes cannot capture, both qualitative and quantitative approaches should be applied to the design or improvement of technologies. Albert and Tullis [44] emphasized the importance of using standardized tools to ensure measurement quality because these tools are valid, reliable, and available for comparison purposes.

We also noted that just 1 study had assessed all the usability attributes defined by the ISO [18]. Most studies assessed elements covering only part of the theoretical concept, with satisfaction and effectiveness being the most common attributes, while other important aspects such as ease of use or safety are left out. This observation is also shared by Sousa and Dunn Lopez [47]. The results of their review indicated that the questionnaires constructed and used to assess usability in studies only assessed part of the usability construct. However, it is possible that some effectiveness studies did not include the assessment of efficiency and satisfaction, as these data could be assessed earlier in the intervention design process.

### Limitations

Although the use of technology in health and exercise has gained widespread popularity in recent years, particularly with the rise of remote interventions, high-quality studies specifically evaluating the usability of these technologies remain limited. This gap raises concerns about the reliability and generalizability of the information currently available and highlights the need for rigorous scientific research focused on usability aspects.

As our review suggests, a reason for the limited number of studies included is that usability testing is often overlooked or insufficiently addressed in research involving older adults. This observation is consistent with the findings of Meiland et al [48], who reported that usability issues related to technologies designed for people with dementia are rarely studied. More broadly, the field of usability testing for remote interventions in aging populations appears to be still emerging, with its relevance becoming more visible during events such as the COVID-19 pandemic [20].

To reflect the current state of the literature, we included pilot studies, which are particularly useful in areas where large-scale evaluations are still lacking. These studies often serve as precursors to broader investigations and provide valuable insights into the feasibility and usability of technological interventions. Pilot studies can assess (1) participant recruitment processes, (2) the reliability and adaptability of technological tools, and (3) potential adverse events—whether related to the technology, the exercise intervention, or user safety [49,50].

In this sense, even preliminary data contributes meaningfully to understanding usability and helps inform the development of future interventions.

### Perspectives

The findings of this review highlight the potential of videoconferencing technologies to support access and continuity of remote physical exercise programs for older adults in home-based settings. This approach is especially useful for individuals who have difficulty traveling, such as those with mobility limitations or who live in remote areas. Beyond healthy older adults, it would be relevant to assess the usability of these technologies in rehabilitation settings, where individuals with troubles often have specific needs. Future studies could explore how cognitive, sensory, or motor impairments may affect interaction with digital tools, and how these technologies may need to be adjusted in terms of usability and support.

To provide a more complete understanding of usability, future research could combine qualitative and quantitative approaches, using mixed methods and standardized tools. This would allow for more consistent and comparable results, reflecting both user and professional perspectives. As remote interventions become more common—especially following the acceleration brought by the COVID-19 pandemic—it is important to continue research on how these technologies are integrated into care practices, making sure they remain acceptable, effective, and safe for all types of users.

### Conclusion

The results of this exploratory study highlight the ease of use of videoconferencing support technologies to deliver remote exercise programs, highlighting positive aspects such as usability, user satisfaction, efficiency, and effectiveness. However, our findings also highlight the importance of environmental support, such as financial assistance and training opportunities, which are essential in helping older people to use information and communication technologies. In the future, it is imperative to conduct research, using standardized tools, to enhance the usability of technologies tailored to the specific needs and characteristics of older adults, thereby ensuring the clinical effectiveness of these interventions.

## Supplementary material

10.2196/65552 Checklist 1 PRISMA-ScR checklist.

## Abbreviations

ISO: International Organization for Standardization
PE: physical exercise
